# The Multifaceted Effects of Serotonin Transporter Polymorphism (5-HTTLPR) on Anxiety, Implicit Moral Attitudes, and Harmful Behaviors

**DOI:** 10.3389/fpsyg.2020.01521

**Published:** 2020-07-10

**Authors:** Róger Marcelo Martínez, Chin-Yau Chen, Tsai-Tsen Liao, Yawei Cheng, Yang-Teng Fan, Shih-Han Chou, Chenyi Chen

**Affiliations:** ^1^Graduate Institute of Injury Prevention and Control, Taipei Medical University, Taipei, Taiwan; ^2^School of Psychological Sciences, National Autonomous University of Honduras, Tegucigalpa, Honduras; ^3^Department of Surgery, National Yang-Ming University Hospital, Yilan, Taiwan; ^4^Graduate Institute of Medical Sciences, College of Medicine, Taipei Medical University, Taipei, Taiwan; ^5^Institute of Neuroscience, Brain Research Center, National Yang-Ming University, Taipei, Taiwan; ^6^Department of Physical Medicine and Rehabilitation, National Yang-Ming University Hospital, Yilan, Taiwan; ^7^Department of Education and Research, Taipei City Hospital, Taipei, Taiwan; ^8^Department of Biological Science and Technology, National Chiao Tung University, Hsinchu, Taiwan; ^9^Department of Physical Medicine and Rehabilitation, Taipei Medical University Hospital, Taipei Medical University, Taipei, Taiwan; ^10^Research Center of Brain and Consciousness, Shuang-Ho Hospital, Taipei Medical University, Taipei, Taiwan; ^11^Graduate Institute of Mind, Brain and Consciousness, College of Humanities and Social Sciences, Taipei Medical University, Taipei, Taiwan; ^12^Psychiatric Research Center, Wan Fang Hospital, Taipei Medical University, Taipei, Taiwan

**Keywords:** 5-HTTLPR, moral judgment, justice sensitivity, implicit moral attitude, moral permissibility

## Abstract

Morality is fundamentally human in nature. Regardless, and even when moral norms seem to work toward the common goal of human cooperation, which morally contentious behaviors are permitted and which are prohibited vary across populations. Because of this occurrence, much scientific debate has revolved around the notion that this phenomenon might be explained by the interaction between genes and environment. Alongside, whether the principles cementing the bases of morality are intuition- or reason-based is another question that has been raised. However, previous research addressing these topics used explicit measures to probe moral attitudes, thus being the participants able to intentionally modify or disguise their honest responses. What’s more, while the 5-HTT gene was found to be associated with anxiety, morality, and even cultural structures, a single genotype–phenotype linkage cannot be established without considering the multifaceted effects of the 5-HTT gene on gene–behavior interactions. In order to explore the role of genetics on modeling moral attitudes and behaviors, we genotyped the 5-HTTLPR in 114 healthy volunteers and subsequently assessed their explicit justice sensitivity (Justice Sensitivity Inventory) and moral permissibility judgments, as well as their implicit moral attitudes [moral implicit association task (mIAT)]. Results revealed that 5-HTTLPR short-allele carriers had significantly lower mIAT reaction times when answering correctly and were less compliant on harming another person even when harm or death would inevitably occur anyway to this other individual. With these preliminary results, we can first see how it does not have to be a matter of vouching for a rationalist versus an intuitionist model of moral judgment, but rather being moral judgment an outcome of the different variants of the 5-HTTLPR polymorphism affecting the way in which individuals engage contrastingly with moral issues.

## Introduction

Morality, independently of how we define it, is fundamentally human in nature (Tomasello and Vaish, 2013). As humanity began coexisting in groups and sharing resources, thus forming societies as a mean for survival, we have found ourselves in the need for sets of moral norms that provide us with protection against perils that can negatively affect our health and integrity, while the moral attitudes and behaviors created by such norms safeguard us from criminal behavior and minimize social conflict (Yoder and Decety, 2018). Nevertheless, even though moral norms, attitudes, and behaviors seem to work toward the common goal of human cooperation, which morally contentious behaviors are permitted and to what extent, and which are prohibited, vary across populations (Mrazek et al., 2013). Such variation on permissibility can be attributed to the interaction between gene inheritance and its expression driven by environmental factors (Marsh et al., 2011; Mrazek et al., 2013; Bernhard et al., 2016; Gong et al., 2017).

The neuromodulator serotonin influences both a wide range of purely physiological tasks and those functions involved with more complex outcomes such as behavior and cognition (Jacobs and Azmitia, 1992; Jonnakuty and Gragnoli, 2008). The 5-HTTLPR is a polymorphism in the promoter region of the serotonin transporter encoding gene, whose two different variants (short and long) have differential effects on the way the serotonin transporter carries out its task, with the short (S) version transporting significantly less serotonin back to the presynaptic neuron when compared to the long (L) version, hence leaving an excess of serotonin in the synaptic cleft to continue stimulating the serotonin receptors (Canli and Lesch, 2007).

Consequently, being an S-allele carrier has been previously associated with anxiety-related traits and neuroticism (Lesch et al., 1996; Mazzanti et al., 1998; Greenberg et al., 2000; Schinka et al., 2004; Sen et al., 2004; Munafò et al., 2005). Moreover, a study (Marsh et al., 2011) yielded findings that being an L-allele carrier may predict having utilitarian moral judgments when compared to S-allele carriers, the latter of whom were observed to possess moral judgments that are to be classified as deontological. Alongside said study, there has been other research supporting the notion that gene inheritance can impact moral attitudes and moral decision-making. For instance, two different studies have observed the influence of the oxytocin receptor gene (OXTR) on moral judgments (Bernhard et al., 2016) and blame attribution (Walter et al., 2012). The first study regarding the OXTR gene observed that those carriers of the T-allele were more likely to exhibit deontological judgments (Bernhard et al., 2016). Conversely, the second study observed that those with the C allele of the OXTR assigned more blame to harm committed accidentally than those non-carriers of said allele (Walter et al., 2012). Another study investigating the CAG polymorphism of the androgen receptor gene (Gong et al., 2017), which regulates testosterone function, yielded the interesting results that the CAG polymorphism modulated moral permissibility to harm only in female subjects. That is, women with more S-allele copies (which translates to higher testosterone availability) were more likely to be permissible toward harmful–utilitarian and unintentional–harmful behaviors, effects that male subjects did not exhibit. Furthermore, and returning to the 5-HTT polymorphism, a study encompassing 21 nations found that those countries with a higher frequency of S-allele carriers among their populations were those countries subject of continuous environmental threat (e.g., diseases, unfavorable weather conditions, etc.) and contained in their territories societies, which evolved and supported cultures where morally contentious behaviors were less permitted, when compared with those nations with higher frequency of L-allele carriers, as resources leading to their survival needed to be controlled more tightly (Mrazek et al., 2013). Interestingly, a study by Brandt and Wetherell (2011) provided strong evidence supporting the notion that the more prone an attitude is to be genetically inherited, the more likely it is to be experienced as moral.

Nevertheless, the aforementioned studies used questionnaires and sets of moral dilemmas as behavioral data for their associations, both of which are self-reported measures that probe explicitly for moral attitudes, thus leaving the veracity of these instruments to the ability of the participants to intentionally modify or disguise their honest responses. As an answer to this issue, besides assessing the relationship between the 5-HTTLPR polymorphism and explicit moral attitudes, this study also utilized the morality version of the Implicit Association Test [moral implicit association task (mIAT)], to assess the individual’s involuntary attitudes to morally laden scenarios (Greenwald et al., 1998; Luo et al., 2006). The mIAT (or every version of the IAT for that matter) rests on the ability of the individual to categorize as quickly as possible two concepts with an attribute (e.g., “moral” vs. “immoral” actions for an evaluation attribute). The easier and quicker the subject forms correct associations between such concepts and the designated attribute, the more strongly are they considered to be associated in memory, in contrast to when it takes more time and it is more difficult for the individual to do so, even when this slower response might also result in a correct pairing. Because of this design, this computer-based test helps reveal attitudes that, because of their underlying, automatic nature, ordinarily exist out of the subject’s scope of awareness (Greenwald et al., 1998).

The objective of this study is to assess the association between implicit attitudes and the genotype variants of the 5-HTTLPR polymorphism, while also considering the multifaceted effects of the microenvironment on gene–behavior interactions, specifically on the permissibility of harmful behaviors. Given the impact of the “epigenetic” mechanisms that encode environmental information from both internal and external bodily sources, a single genotype–phenotype linkage cannot be established without simultaneously considering the multifaceted effects of the 5-HTT gene, which has been found to be associated with anxiety, morality, and even culture structures (Fergusson et al., 2011; Mrazek et al., 2013; Perkins et al., 2013; Yang et al., 2019). Furthermore, the present study can also help us shed some light on the well-known argument of affect and cognition in regard to morality. It is plausible to see how it does not have to be a matter of choosing to vouch for a rationalist model (Piaget, 1932; Kohlberg, 1984) versus an intuitionist model (Haidt, 2001) of moral judgment, but rather being both two different outcomes of the same model, where moral judgment appears as the product of the interaction between environmental factors and the different variants of the 5-HTTLPR polymorphism, and affecting the way in which individuals engage contrastingly with moral issues.

## Materials and Methods

### Participants

This study was part of the social neuroscience project: *From Genetic Heterogeneity and Brain Connectome to Social Neuroscience (YM104078E)*, carried out in the National Yang-Ming University, which investigates the individual difference in genetic heterogeneity, anxiety, moral attitudes, brain structure and functions, and their relations with the neuropharmaceutical drug: lorazepam (2016/10/11 to 2018/09/30). During this period, we evaluated 567 adults [(male-to-female ratio, 266/301), aged between 18 and 63 years (25.66 ± 8.19 years)], who were recruited from the general population using an online survey disseminated through social media and were having their first appointment in the Social Neuroscience Laboratory. The genetic part of this study comprised 114 adults (male-to-female ratio, 53/61), aged between 18 and 46 years (23.4 ± 4.03 years). All participants were Han Chinese and right-handed. They participated in the study after providing written informed consent and were screened for major psychiatric illnesses (e.g., general anxiety disorder) by the Structured Clinical Interview for *Diagnostic and Statistical Manual of Mental Disorders, Fourth Edition* Axis I Disorders, and excluded if there was evidence of comorbid neurological disorders (e.g., dementia, seizures), history of head injury, and alcohol or substance abuse or dependence within the past 5 years. All participants had normal vision. The subjects were included in the data analysis and subdivided into three groups on the basis of their genotyping results; participants possessing one copy of the S allele and one copy of the L allele were included in the L/S group, and those homozygous for the S or L allele were included in the S/S or L/L group, respectively. This study was approved by the ethics committee of the National Yang-Ming University Hospital and conducted in accordance with the Declaration of Helsinki.

### DNA Extraction and 5-HTTLPR Genotyping

Buccal cells were harvested from the inner cheek of each subject to provide DNA for genetic testing. The DNA was extracted from buccal swabs using a QIAamp DNA Mini Kit. The procedure employed a polymerase chain reaction (PCR)–based protocol followed by restriction endonuclease digestion to identify the 5-HTTLPR that are located in the promoter region of the serotonin transporter gene (*SLC6A4*) and rs25531 variants: S, LA, and LG. Forward primer: 5′-TCCTCCGCTTTGGCGCCTCTTCC-3′ and reverse primer: 5′-TGGGGGTTGCAGGGGAGATCCT-3′ (10 μM each) were used for 50 μL PCR containing approximately 25 ng DNA, 25 μL Taq DNA Polymerase 2× Master Mix Red (Ampliqon) and ddH2O, with an initial 5-min denaturation step at 95°C followed by 35 PCR cycles of 95°C (30 s), 65°C (40 s), and 72°C (30 s) and a final extension step of 5 min at 72°C. To distinguish the A/G single-nucleotide polymorphism of the rs25531, we extracted 10 μL of the PCR product for digestion by FastDigest *Hpa*II (Thermo, FD0514), an isoschizomer of *Msp*I, a total reaction of 20 μL. These were loaded side by side on 2.5–3.0% agarose gel.

### General Procedures

In this study, we used the Justice Sensitivity Inventory (JSI), mIAT, and moral dilemma to assess dispositional justice sensitivity, implicit moral attitudes, and moral permissibility to harm, respectively. Self-reported anxiety levels were determined using the State-Trait Anxiety Inventory (STAI) (Spielberger et al., 1970). Participants completed these tasks in a counterbalanced order after sampling their buccal cells.

### Dispositional Justice Sensitivity

The JSI is a self-report psychometric measure that assesses four perspectives of justice sensitivity (them being victim, beneficiary, observer, and perpetrator justice sensitivity) and produces four scores between 1 and 7, which index an individual’s disposition to react to unfair situations. Each perspective has 10 items, which are rated on a seven-point scale from 0 (not at all) to 7 (strongly agree). The scores indicate an individual’s perceptual threshold of moral norm violation and injustice. The closely related subfactors of beneficiary, observer, and perpetrator sensitivity are collapsed to create a measure of other-oriented sensitivity (Edele et al., 2013). Although related, self-orientation and other-orientation represent reliably distinct constructs that can exert independent and opposing influences on behavior (Gollwitzer et al., 2009). Self-orientation tends be associated with higher neuroticism and lower agreeableness, whereas other-oriented justice sensitivity is related to high agreeableness, conscientiousness, and empathy (Schmitt et al., 2010).

### Implicit Moral Attitude (mIAT)

The mIAT was modified with animations and words for the stimuli. The verbal stimuli included 26 extremely pleasant and 26 extremely unpleasant words, selected from high frequently used Chinese words (Chen et al., 2002). The animation stimuli comprised 47 clips depicting everyday dyadic interactions. In each animation, the action is characterized as either a moral or immoral action based on an outcome of personal assistance or harm (Yoder and Decety, 2014a, b). The mIAT followed the experimental design proposed by Greenwald et al. (1998), including the five discrimination blocks:

Block 1 starts with the “initial target-concept discrimination.” Participants categorize the clips as moral (right response key) or immoral (left response key).
Block 2 is termed as “attribute discrimination.” Participants categorized words as negative (right response key) or positive (left response key).
Block 3 “target-concept discrimination” combines Blocks 1 and 2 with clips and words randomly presented in alternative trials. The moral clips share a right response key with negative words, and immoral clips share a left response key with positive words (moral-negative/immoral-positive).
Block 4 is known as “reversed target-concept discrimination.” Participants learn a reversed response assignment for Block 1 and judged if clips are moral (left response key) or immoral (right response key).
Block 5 combines Blocks 2 and 4. The immoral clips share a right response key with negative words, and moral clips share a left response key with positive words (immoral-negative/moral-positive).

Two block sequences (12345/42513) were counterbalanced to control for the sequential effect. Half of the participants experienced the incongruent block first, and the other half completed the congruence block first. Blocks 1, 2, and 4 contained 20 trials each, whereas Blocks 3 and 5 (incongruent and congruent blocks) contained 40 trials each. Words and/or clips within each block were presented in a random order. Each trial consisted of a 1,000-ms presentation of a fixation cross followed by a stimulus. The stimuli were viewed on a computer and administered using E-Prime version 2.0 software (Psychology Software Tools). Participants were instructed to classify each word or clip as fast and accurately as possible. There were 10 practice trials before participants started Block 3 or 5. The accuracy rate and reaction time (RT) during Blocks 3 and 5 were recorded.

The mIAT performance, as indexed by *D* scores, represents the implicit moral attitude. The *D* score was calculated by subtracting the mean RT of congruent (immoral-negative) blocks from that of incongruent (immoral-positive) blocks and dividing it by the pooled standard deviation across the two blocks (Nosek et al., 2014). The higher *D* scores might come from higher RTs for the incongruent blocks, or lower RTs for the congruent blocks. Because the IAT relies on the RT differentials, which are highly sensitive to outliers and extreme values, reaction times exceeding two times the standard deviation from the subject means were excluded from the set of valid responses (outliers accounted for <3% of all the responses). Additionally, extreme responses—either very slow or very fast—can indicate inattention to the task performance rules. Exclusion criteria were applied for RTs faster than 300-ms and slower than 3,000-ms cutoff boundary response latency (extreme responses accounted for <1% of all responses) (Nosek et al., 2014).

### Moral Dilemma Task

Based on previous work (Greene et al., 2001, 2004, 2009; Huebner et al., 2011)^1^, 48 moral dilemmas were selected in order to make two versions of the moral judgment task balanced on emotional intensity (Koenigs et al., 2007). Each version consists of 24 dilemmas with nine non-moral dilemmas (e.g., driving a turnip harvesting machine, and depending on whether you choose the left path or the right path, you will harvest 10 or 20 bushels of turnips, respectively), five impersonal dilemmas, and 10 personal dilemmas. The impersonal dilemmas involve indirect harm (e.g., flipping a switch, as in the trolley dilemma), whereas personal dilemmas include harm through direct physical contact. The personal dilemmas are further divided into dilemmas in which the death of or harm to the victim is inevitable (e.g., Rescue 911 dilemma, described below in the discussion), or evitable (e.g., pushing a stranger to his death to save other people’s lives, as with the footbridge dilemma). Moral permissibility judgments are higher for transgressions that lead to inevitable harm by the principle of lesser evil (Hauser, 2006; Mikhail, 2007). Dilemmas were translated from English to Chinese and then translated back from Chinese to English and checked for consistency by a native English speaker. Participants read and responded to the dilemmas at their own pace.

## Results

### Dispositional Traits and Morality

**FIGURE 1:**
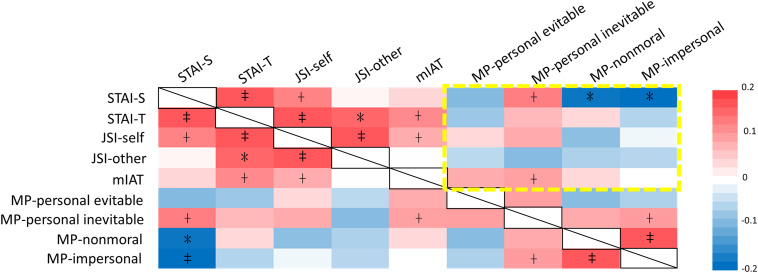
Significance plot of the intercorrelations among dispositional measures of anxiety, justice sensitivity, implicit moral attitudes, and moral permissibility. State anxiety was positively correlated with the permissibility of inevitable harm, but negatively correlated with non-moral and impersonal harm. Implicit moral attitude (mIAT) was positively correlated with the permissibility of inevitable harm. The colors and color bar values (right) represent the Pearson correlation coefficient (For the exact values, see Supplementary Table 1). STAI, state-trait anxiety inventory; JSI, justice sensitivity inventory; mIAT, moral implicit association test; MP, moral permissibility; + *p* < 0.1; *p < 0.05; ‡*p* < 0.001.

Intercorrelations among measures of anxiety, dispositional justice sensitivity, implicit moral attitudes, and moral permissibility were first inspected to identify the potential contributing factors that could modulate moral permissibility of harm (Figure 1 and Supplementary Table 1). Significant factors (*p* < 0.05, one-tailed) were further included into a univariate general linear model to examine the true effect of the 5HTT polymorphism on the dilemmatic moral decisions (Table 1). State anxiety was positively correlated with the permissibility of inevitable harm, but negatively correlated with non-moral and impersonal harm. Implicit moral attitudes (mIAT) were positively correlated with the permissibility of inevitable harm.

**TABLE 1:** Results of the univariate general linear model based on the permissibility of personal inevitable harm as dependent variable and age, gender, STAI-S, mIAT, and 5-HTTLPR polymorphism as independent variables.

Independent variable	Model (MP-personal inevitable harm)			
	*df*	*F*	*p*	η^2^
Corrected Model	5	2.376	0.044	0.1
Intercept	1	0.247	0.620	0.002
Gender	1	1.632	0.204	0.015
Age	1	1.605	0.208	0.015
STAI-S	1	0.657	0.419	0.006
mIAT	1	1.558	0.215	0.014
5-HTTPLPR genotype	1	4.034	0.047	0.036

### Genotyping Distribution

5-HTTLPR polymorphism was found to have allele frequencies of S, *n* = 163 (71.5%); LA, *n* = 24 (10.5%); and LG, *n* = 41 (18%), and a genotype distribution of S/S, *n* = 62 (54.4%); S/LG, *n* = 23 (20.2%); LG/LG, *n* = 6 (5.3%); S/LA, *n* = 16 (14%); LG/LA, *n* = 6 (5.2%); and LA/LA, *n* = 1 (0.9%). Genotype distribution of the 5-HTTLPR across all participants was in Hardy–Weinberg equilibrium, χ^2^(3) = 3.84, *p* = 0.28. The following analyses employed the genotype groups: L/L = 13, L/S = 39, and S/S = 62. 5-HTTLPR polymorphism did not affect age (L/L, L/S, S/S, mean ± SE: 22.08 ± 0.6 vs. 24.51 ± 0.73 vs. 22.98 ± 0.49; *p* = 0.1) and gender (L/L, L/S, S/S, male (% of total): 54% vs. 49% vs. 44%; *p* = 0.8).

### Dispositional Justice Sensitivity Results

**FIGURE 2:**
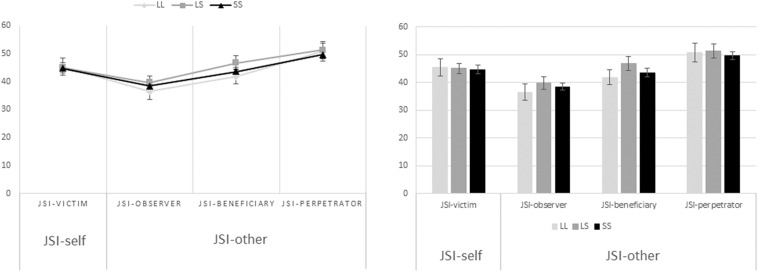
Association between the 5-HTTLPR genotype groups and self-oriented and other-oriented justice sensitivity. In the one-way ANOVA performed to assess the relationship between the serotonin transporter genotype groups (L/L, L/S, S/S), and self-oriented (46 ± 2.95, 46 ± 1.62, 44.6 ± 1.36, mean ± SE, respectively) and other-oriented (43.5 ± 2.51, 46 ± 1.80, 44.6 ± 1.05, mean ± SE, respectively) justice sensitivity, the results yielded no significant differences between group means neither when it comes to self-oriented [*F*(2,111) = 0.245, *p* = 0.783], nor when it comes to other-oriented justice sensitivity [*F*(2,111) = 0.399, *p* = 0.672]. In the same manner, the MANCOVA performed to assess the same relationship but controlling for age and gender was also non-significant [*F*(4,216) = 0.391, *p* = 0.815, Wilks’ Λ = 0.986, ηp^2^ = 0.007].

A one-way analysis of variance (ANOVA) was performed as to assess the relationship between the 5-HTTLPR genotype groups (L/L, L/S, S/S) and self-oriented (46 ± 2.95, 46 ± 1.62, 44.6 ± 1.36, mean ± SE, respectively) and other-oriented (43.5 ± 2.51, 46 ± 1.80, 44.6 ± 1.05, mean ± SE, respectively) justice sensitivity (Figure 2). The results yielded no significant differences between group means neither when it comes to self-oriented justice sensitivity [*F*(2,111) = 0.245, *p* = 0.783] nor when it comes to other-oriented justice sensitivity [*F*(2,111) = 0.399, *p* = 0.672]. Furthermore, a one-way multivariate analysis of covariance (MANCOVA) was performed to assess the exact same relationship between variables but controlling for age and gender. Nevertheless, results remained non-significant when it comes to mean differences between the genotype groups on the combined dependent variables after controlling for the aforementioned covariates [*F*(4,216) = 0.391, *p* = 0.815, Wilks’ Λ = 0.986, ηp^2^ = 0.007].

### Implicit Moral Attitude (mIAT) Results

**FIGURE 3:**
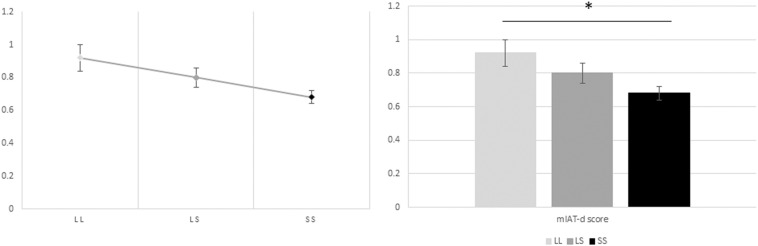
Relationship between the genotype groups and the *D* scores obtained from the mIAT. The one-way ANOVA yielded a statistically significant difference between genotype group mean *D* scores [*F*(2,111) = 3.432, *p* = 0.036]. *Post hoc* analyses revealed that *D* scores were significantly lower in those homozygous for the S allele (0.68 ± 0.042, mean ± SE, *p* = 0.024) when compared to those homozygous for the L allele (0.91 ± 0.071, mean ± SE, *p* = 0.024). There was no statistically significant difference, however, between the heterozygous (L/S)(0.80 ± 0.058, mean ± SE) and the homozygous (S/S and L/L) groups of alleles (*p* = 0.069 and *p* = 0.318, respectively). The subsequent ANCOVA showed that the difference between genotype group mean *D* scores remained statistically significant also when controlling by age and gender [*F*(2,109) = 3.242, *p* = 0.043, ηp^2^ = 0.056]. *Post hoc* analyses further underlined the statistically significant difference between the lower mean *D* scores among those homozygous for the S allele and the higher *D* scores among those homozygous for the L allele (*p* = 0.024), whereas the difference in means between those with heterozygous alleles (L/S) and those with homozygous alleles (S/S and L/L) remained non-significant (*p* = 0.102 and *p* = 0.276, respectively). Likewise, the linear regression analysis exhibited a statistically significant linear association between genotype group (number of risk allele “S”) and mIAT *D* scores [*F*(3,110) = 2.514, *p* = 0.012, *R*^2^ = 0.064], and controlling for age and gender. Accordingly, there is a decrease of 0.117 points in the participants’ *D* scores as a function of a one increase in S alleles being carried.

Subsequently, a one-way ANOVA was also performed to assess the relationship between genotype groups and *D* scores obtained from the mIAT. The results yielded a statistically significant difference between genotype group mean *D* scores [*F*(2,111) = 3.432, *p* = 0.036]. *Post hoc* analyses revealed that *D* scores were significantly lower in those homozygous for the S allele (0.68 ± 0.042, mean ± SE, *p* = 0.024) when compared to those homozygous for the L allele (0.91 ± 0.071, mean ± SE, *p* = 0.024) (Figure 3). There was no statistically significant difference, however, between the heterozygous (L/S)(0.80 ± 0.058, mean ± SE) and the homozygous (S/S and L/L) groups of alleles (*p* = 0.069 and *p* = 0.318, respectively).

Moreover, a one-way analysis of covariance (ANCOVA) was performed as to again control for age and gender. The analysis results showed that the difference between genotype group mean *D* scores remained statistically significant also when controlled by the aforementioned covariates [*F*(2,109) = 3.242, *p* = 0.043, ηp^2^ = 0.056]. *Post hoc* analyses further underlined the statistically significant difference between the lower mean *D* scores among those homozygous for the S allele and the higher *D* scores among those homozygous for the L allele (*p* = 0.024), whereas the difference in means between those with heterozygous alleles (L/S) and those with homozygous alleles (S/S and L/L) remained non-significant (*p* = 0.102 and *p* = 0.276, respectively).

Furthermore, when performing a linear regression analysis, and controlling for age and gender, there appears to be a statistically significant linear association when the genotype group (number of risk allele “S”) is included as the independent variable, and mIAT *D* scores are used as the dependent variable [*F*(3,110) = 2.514, *p* = 0.012, *R*^2^ = 0.064]. These results show there is a decrease of 0.117 points in the participants’ *D* scores as a function of a one increase in S alleles being carried.

### Moral Permissibility of Harmful Behavior in Moral Dilemma Task

**FIGURE 4:**
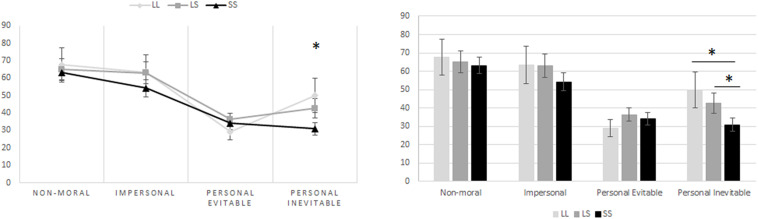
S-allele carriers of the 5-HTTLPR polymorphism exhibited deontological moral judgments for personal inevitable moral dilemmas. The one-way ANOVA revealed that genotype group mean differences were statistically significant for personal moral dilemmas where harm or death to another individual is inevitable [*F*(2,111) = 3.196, *p* = 0.045]. *Post hoc* analyses further showed that in the personal inevitable moral dilemmas, those homozygous for the S-allele (34.27 ± 3.55, mean ± SE, *p* = 0.048) were less compliant on harming another person, even when harm or death would inevitably occur to this other individual, when compared to those homozygous for the L allele (51.92 ± 9.16, mean ± SE, *p* = 0.048). In the same manner, the difference between the means of those homozygous for the S allele and those with heterozygous alleles (L/S) (46.15 ± 4.64, mean ± SE, *p* = 0.047) was also significant. Nevertheless, there was no statistically significant difference between the means of those with heterozygous alleles (L/S) and those with homozygous L alleles (*p* = 0.535). Furthermore, there is a statistically significant linear association between genotype group (number of risk allele “S”) and personal inevitable moral dilemmas, when controlling for age and gender [*F*(3,110) = 3.256, *p* = 0.019, *R*^2^ = 0.082]. Hence, there is a 9.27% decrease in the participants’ willingness to answer positively to perform an action that would have as an outcome harm or death (even when such harm or death is in the end inevitable) to another person, as a function of a one increase in S alleles being carried.

When it comes to the association between the genotype groups of the 5-HTTLPR and the moral dilemmas, the one-way ANOVA revealed that genotype group mean differences were statistically significant when it comes to personal moral dilemmas where harm or death to another individual is inevitable [*F*(2,111) = 3.196, *p* = 0.045]. However, there were no statistically significant differences among the other dilemmas, these being non-moral dilemmas [*F*(2,111) = 0.318, *p* = 0.728], impersonal moral dilemmas [*F*(2,111) = 0.419, *p* = 0.658], and personal moral dilemmas where harm or death to another individual is evitable [*F*(2,111) = 0.089, *p* = 0.915]. *Post hoc* analyses further showed that in the personal moral dilemmas with inevitable harm or death, those homozygous for the S allele (34.27 ± 3.55, mean ± SE, *p* = 0.048) were less compliant on harming another person, even when harm or death would inevitably occur to this other individual, when compared to those homozygous for the L allele (51.92 ± 9.16, mean ± SE, *p* = 0.048). In the same manner, the difference between the means of those homozygous for the S allele and those with heterozygous alleles (L/S) (46.15 ± 4.64, mean ± SE, *p* = 0.047) was also significant. Nevertheless, there was no statistically significant difference between the means of those with heterozygous alleles (L/S) and those with homozygous L alleles (*p* = 0.535) (Figure 4). However, when performing the MANCOVA as to include gender and age as covariates in the model, the association between the genotype group on the combined dependent variables’ means became non-significant [*F*(8,212) = 1.103, *p* = 0.362, Wilks’ Λ = 0.922, ηp^2^ = 0.040]. Nevertheless, there appears to be a statistically significant linear association when the genotype group (number of risk allele “S”) is used as the independent variable, and the responses to personal moral dilemmas with inevitable harm or death are included as the dependent variable in a linear regression model controlling for age and gender [*F*(3,110) = 3.256, *p* = 0.019, *R*^2^ = 0.082]. These results showed a 9.27% decrease in the participants’ willingness to answer positively to perform an action that would have as an outcome harm or death (even when such harm or death is in the end inevitable) to another person, as a function of a one increase in S alleles being carried. A univariate general linear model, based on the permissibility of personal inevitable harm as dependent variable, and age, gender, STAI-S, mIAT, and 5HTTLPR polymorphism as independent variables, was applied to examine the effect of the 5HTT polymorphism. The 5HTT genotype remained significant after controlling for the STAI-S and mIAT (Table 1).

## Discussion

The aim of the present study was mainly that of assessing implicit attitudes in relation to the genotype variants of the 5-HTTLPR polymorphism, while also considering the multifaceted effects of the microenvironment on gene–behavior interactions, specifically on the permissibility of harmful behaviors. This would help us tackle the well-known and widely debated argument of affect versus cognition in regard to moral decision-making. Given the impact of the “epigenetic” mechanisms that encode environmental information from both internal and external bodily sources, a single genotype–phenotype linkage cannot be established without simultaneously considering the multifaceted effects of the 5-HTT gene, which has also been found to be associated with anxiety, morality, and even cultural structures (Fergusson et al., 2011; Mrazek et al., 2013; Perkins et al., 2013; Yang et al., 2019). While anxiety was found to be significantly associated with the permissibility to harm, the direction of such correlation was opposite, depending on the emotional valence and moral justification behind the harmful behaviors. When a harmful action was performed for the noble goal of benefitting the majority, even though such action bore an inevitable sacrifice, individuals with higher anxiety were found to release themselves and show more willingness to become the driving force of this type of harmful behaviors. However, when the harm was not inevitable and could be explicitly attributed to the agents, individuals with higher anxiety were reluctant to administer it. Implicit moral attitudes, which refer to the immediate, unintentional tendency or inclination toward moral correctness and to deviate from wrongness of actions, were also found positively correlated with the permissibility of inevitable harm. However, the 5-HTT genotype remained significant after controlling for both the STAI-S and mIAT scores. These results are supported by the Differential Susceptibility Hypothesis (Belsky, 2016), which explains how individuals experience life circumstances and events in a differing manner, dependent on preexisting biological factors, and which may result in certain predispositions.

Furthermore, the analyses revealed that the *D* scores were significantly lower in those homozygous for the S allele when compared to those homozygous for the L allele, even when controlled for age and gender. In the association between the polymorphism and the moral dilemmas, those homozygous for the S allele were less compliant on harming another person, even when harm or death would inevitably occur anyway to this other individual, relative to those homozygous for the L allele. In regard to justice sensitivity, the results yielded no significant differences between group means neither when it comes to self-oriented justice sensitivity nor when it comes to other-oriented justice sensitivity.

The S-allele carriers obtained lower *D* scores than those homozygous for the L allele. A lower *D* score reflects lower RTs when performing the mIAT; thus, the S-allele carriers were better able to switch rapidly between tasks and respond accordingly. It is possible this effect is due to two mechanisms. The first is in regard to increased amygdala reactivity, which consequently heightens vigilance (Canli and Lesch, 2007). Because the amygdala is implicated in the automatic evaluation of socially relevant stimuli and expression of implicit attitudes (Phelps et al., 2000), it is reasonable to infer that S-allele carriers are overpowered by such implicit processes and rely more in moral intuitions (Feinberg et al., 2012), whereas L-allele carriers are able to override them, thus being able to have enough cognitive capacity to reason through the task (Lucas and Galinsky, 2015), albeit consequently slowing their reaction times, as it has also been seen that those homozygous for the L allele can have an enlarged Dorsolateral Prefrontal Cortex (dlPFC), although this research observed this phenomenon on emotionally neglected children (Frodl et al., 2010). Second, the mechanism can be due to environmental constraints, as the hypervigilance of which S-allele carriers are subject to makes them more attentive to tasks and even to environmental dangers. A study encompassing 21 nations revealed that those societies with a higher frequency of S-allele carriers among their population accepted less morally contentious behavior; thus, the society was characterized as being culturally tight. Interestingly, this phenomenon correlated with the ecological threat these nations were or are subject to. In other words, the more ecological threat the nation had been through in history, hence, the more resources it needed to take care of in order to survive, the less these societies justified morally deviant behavior, finding which correlated positively with increments in the amount of S-allele carriers among the different countries’ populations (Mrazek et al., 2013). In the same vein, although justice sensitivity was not found to be significantly associated with the permissibility of harm, it was found to be modulated by anxiety and implicit moral attitudes, both of which were intercorrelated with the 5-HTT polymorphism. These results show ecological and gene–environmental interactions and help explain why Han Chinese participants exhibit a higher proportion of the S/S genotype and a lower frequency of the L/L genotype than that observed in Caucasian populations, where the S allele was seen as conferring less susceptibility toward anxiety in Asian people (Chen et al., 2020).

In regard to moral dilemmas and dispositional justice sensitivity, there were no significant differences between groups in neither of both endeavors. This is very likely due to their nature as self-reported measures assessing explicit—thus, direct and consciously controlled—cognition (Greene et al., 2001, 2004, 2009; Schmitt et al., 2010; Huebner et al., 2011), which can be easily falsifiable depending on context and expectations (Greenwald and Banaji, 1995). The one subscale exhibiting a significant difference, though, was that of personal-inevitable moral dilemmas in the moral dilemma task, where those homozygous for the S allele showed lower scores, hence being less likely to endorse harm or death to another person—than those homozygous for the L allele. The personal-inevitable moral dilemmas subsection is composed of moral predicaments, which in order to be solved, an action entailing direct physical contact (personal)—such as pushing a stranger into some train tracks—is required, and such harm or death to the victim is inevitable, independently of the responder deciding to act or not. One example of a personal-inevitable moral dilemma is that of the “Rescue 911 dilemma”: the participant imagines himself in the place of the leading character, who is flying in a helicopter that suffers a technical error; because of this issue, the participant needs to decide if he throws off one of the patients, the helicopter is carrying as to lighten the aircraft and be able to continue on its way. If the participant decides not to make the sacrifice, however, the helicopter would fall, and absolutely all of its passengers would die anyway—including the patient who was decided was not going to be thrown (Christensen et al., 2014). An explanation for why this was the only scale with a significant difference between those homozygous for the S allele and the homozygous for the L allele, even when the answers can be falsified, might be due to the differential way in which emotions arise between these two groups of participants. While studies have shown that personal moral dilemmas in general elicit increased emotional arousal than impersonal moral dilemmas—with the majority of the participants choosing the deontological option (Christensen et al., 2014; Zhao et al., 2016)—when it comes to personal-inevitable moral dilemmas, participants tend to choose the utilitarian option (Huebner et al., 2011; Christensen et al., 2014), this due to the principle of lesser evil (Hauser, 2006; Mikhail, 2007). In the case of the S-allele carriers, we can entertain the possibility that the aforementioned emotional arousal elicited by personal moral dilemmas as a whole is increased even further by the amygdala hyperreactivity in which S-allele carriers incur (Canli et al., 2005; Canli and Lesch, 2007), thus driving these subjects to choose deontological responses to the personal moral dilemmas even in inevitable circumstances. To explain this point further, we have to take into account that utilitarian moral judgments are driven by the same underlying mechanisms as risky and uncertain choices—defining these as those choices made without any knowledge of what the consequences might be (Kahneman and Tversky, 1984)—this is due to the fact that while the outcome of the deontological options in the moral dilemmas task is clearly known, in the utilitarian options the psychological burden (e.g., how intense or for how long guilt and regret will last) due to choosing such actions is not clear; hence, compared to the deontological choice, the utilitarian option is analogous to risk and uncertainty, two situations that S-allele carriers seem to avoid. In fact, Lucas and Galinsky (2015) posit that it is the downregulation of negative affect (either by blunting emotional reactivity or by facilitating strategies for emotion regulation) that promotes utilitarian and risky choices and that it is the upregulation of such negative affect (which serotonergic activity produces) that inhibits utilitarian preferences. Moreover, theoretically based models of choice under uncertainty propose that risky gambles and utilitarian options are chosen by people when the rewards offered are enough to suppress uncertainty-related anxiety, or when people possess enough cognitive capacity so that moral affects and affective reactions are overridden, because this makes them more capable of reasoning through the moral decision-making process (Greene et al., 2001; Loewenstein et al., 2001).

Extant literature lends evidence supporting this dual-process model, where utilitarian choices solely recruit cognitive resources, are diminished by cognitive load, and are stimulated by the necessity for cognition (Greene et al., 2008; Conway and Gawronski, 2013). Concerning emotion regulation, studies have found that suppressing emotions increase utilitarian preferences (Lee and Gino, 2015) and that emotion reappraisal heightens considerations and decreases the influence of moral intuitions (Feinberg et al., 2012). These different studies evince the two distinct pathways—cognitive and affective—by which the effect of negative moral affect can be modulated, evidence that is also in line with the differences between the variants of the polymorphism in regard to the *D* score.

However, some limitations of this study should be mentioned. First, several studies have observed a significant interaction between the 5-HTT polymorphism and early-life environment on the modulation of behavior and psychological factors (including education) (Assary et al., 2018), which this study did not consider. Nevertheless, this study makes use of a portion of the same sample, and utilizing the same procedures, as other studies (Chen et al., 2020). Second, the sample for those homozygous for the L allele is quite small; thus, replication studies with a greater sample size are warranted. It is noteworthy, however, that Asian populations have been observed to have a greater proportion of S-allele carriers and a lower frequency of L-carriers than other populations (Goldman et al., 2010).

In conclusion, our research suggests that it is not about vouching between a rationalist (Piaget, 1932; Kohlberg, 1984) versus an intuitionist model (Haidt, 2001) of moral judgment, but rather there is a dynamic interplay between cognition and affect. Nevertheless, the outcome of this interplay is not the same across all individuals, and it differs depending on the variants of the 5-HTTLPR polymorphism each person possesses and its interaction with environmental factors. Those with the S allele depend more on moral intuitions by default, even when they later can choose to change their attitudes depending on context and expectation, whereas L-allele carriers exhibit decreased emotional influence on their moral attitudes and judgments, as they recruit more cognitive resources for this purpose. Thus, moral judgment is an outcome that changes depending of the variants of the 5-HTTLPR polymorphism, affecting the way in which individuals engage contrastingly with moral issues.

## Data Availability Statement

The raw data supporting the conclusions of this article will be made available by the authors, without undue reservation, to any qualified researcher.

## Ethics Statement

The studies involving human participants were reviewed and approved by National Yang-Ming University Ethics Committee. The patients/participants provided their written informed consent to participate in this study.

## Author Contributions

CC and YC conceived and designed the study. RM did the literature collection and wrote the first draft. RM and CC performed the statistical analyses. T-TL and C-YC performed the genetic analyses. All authors contributed to the revision of the final draft.

## Conflict of Interest

The authors declare that the research was conducted in the absence of any commercial or financial relationships that could be construed as a potential conflict of interest.

## Footnotes

1 http://www.cell.com/neuron/supplemental/S0896-6273(04)00634-8

## References

[B1] Assary E. Vincent J. P. Keers R. Pluess M. (2018). Gene-environment interaction and psychiatric disorders: review and future directions. *Semin. Cell Dev. Biol.* 77 133–143. 10.1016/j.semcdb.2017.10.016 29051054

[B2] Belsky J. (2016). The differential susceptibility hypothesis: sensitivity to the environment for better and for worse. *JAMA Pediatr.* 170 321–322. 26831915 10.1001/jamapediatrics.2015.4263

[B3] Bernhard R. M. Chaponis J. Siburian R. Gallagher P. Ransohoff K. Wikler D. (2016). Variation in the oxytocin receptor gene (OXTR) is associated with differences in moral judgment. *Soc. Cognit. Affect. Neurosci.* 11 1872–1881. 27497314 10.1093/scan/nsw103 PMC5141955

[B4] Brandt M. J. Wetherell G. A. (2011). What attitudes are moral attitudes? The case of attitude heritability. *Soc. Psychol. Personal. Sci.* 3 172–179. 10.1177/1948550611412793

[B5] Canli T. Lesch K. P. (2007). Long story short: the serotonin transporter in emotion regulation and social cognition. *Nat. Neurosci.* 10 1103–1109. 10.1038/nn1964 17726476

[B6] Canli T. Omura K. Haas B. W. Fallgatter A. Constable R. T. Lesch K. P. (2005). Beyond affect: a role for genetic variation of the serotonin transporter in neural activation during a cognitive attention task. *Proc. Natl. Acad. Sci. U.S.A.* 102 12224–12229. 10.1073/pnas.0503880102 16093315 PMC1189322

[B7] Chen C. Martinez R. M. Liao T. T. Chen C. Y. Yang C. Y. Cheng Y. (2020). An integrative analysis of 5HTT-mediated mechanism of hyperactivity to non-threatening voices. *Commun. Biol.* 3:113. 10.1038/s42003-020-0850-3 PMC7064530 32157156

[B8] Chen H.-C. Li W.-C. Chou T.-A. Cho S.-L. (2002). Measuring the self-exposure of Chinese people using the implicit association test. *Psychol. Test.* 49 217–234.

[B9] Christensen J. F. Flexas A. Calabrese M. Gut N. K. Gomila A. (2014). Moral judgment reloaded: a moral dilemma validation study. *Front. Psychol.* 5:607. 10.3389/fpsyg.2014.00607 25071621 PMC4077230

[B10] Conway P. Gawronski B. (2013). Deontological and utilitarian inclinations in moral decision making: a process dissociation approach. *J. Pers. Soc. Psychol.* 104 216–235. 10.1037/a0031021 23276267

[B11] Edele A. Dziobek I. Keller M. (2013). Explaining altruistic sharing in the dictator game: the role of affective empathy, cognitive empathy, and justice sensitivity. *Learn. Ind. Diff.* 24 96–102. 10.1016/j.lindif.2012.12.020

[B12] Feinberg M. Willer R. Antonenko O. John O. P. (2012). Liberating reason from the passions: overriding intuitionist moral judgments through emotion reappraisal. *Psychol. Sci.* 23 788–795. 10.1177/0956797611434747 22636202

[B13] Fergusson D. M. Horwood L. J. Miller A. L. Kennedy M. A. (2011). Life stress, 5-HTTLPR and mental disorder: findings from a 30-year longitudinal study. *Br. J. Psychiatry* 198 129–135. 10.1192/bjp.bp.110.085993 21282783 PMC3031653

[B14] Frodl T. Reinhold E. Koutsouleris N. Donohoe G. Bondy B. Reiser M. (2010). Childhood stress, serotonin transporter gene and brain structures in major depression. *Neuropsychopharmacology* 35 1383–1390. 10.1038/npp.2010.8 20147891 PMC3055341

[B15] Goldman N. Glei D. A. Lin Y. H. Weinstein M. (2010). The serotonin transporter polymorphism (5-HTTLPR): allelic variation and links with depressive symptoms. *Depress Anxiety* 27 260–269. 10.1002/da.20660 20196101 PMC2841212

[B16] Gollwitzer M. Rothmund T. Pfeiffer A. Ensenbach C. (2009). *Why and When Justice Sensitivity Leads to Pro- and Antisocial Behavior.* Amsterdam: Elsevier Science.

[B17] Gong P. Fang P. Yang X. Ru W. Wang B. Gao X. (2017). The CAG polymorphism in androgen receptor (AR) gene impacts the moral permissibility of harmful behavior in females. *Psychoneuroendocrinology* 80 74–79. 10.1016/j.psyneuen.2017.03.008 28324702

[B18] Greenberg B. D. Li Q. Lucas F. R. Hu S. Sirota L. A. Benjamin J. (2000). Association between the serotonin transporter promoter polymorphism and personality traits in a primarily female population sample. *Am. J. Med. Genet.* 96 202–216. 10.1002/(sici)1096-8628(20000403)96:2<202::aid-ajmg16>3.0.co;2-j 10893498

[B19] Greene J. D. Cushman F. A. Stewart L. E. Lowenberg K. Nystrom L. E. Cohen J. D. (2009). Pushing moral buttons: the interaction between personal force and intention in moral judgment. *Cognition* 111 364–371. 10.1016/j.cognition.2009.02.001 19375075

[B20] Greene J. D. Morelli S. A. Lowenberg K. Nystrom L. E. Cohen J. D. (2008). Cognitive load selectively interferes with utilitarian moral judgment. *Cognition* 107 1144–1154. 10.1016/j.cognition.2007.11.004 18158145 PMC2429958

[B21] Greene J. D. Nystrom L. E. Engell A. D. Darley J. M. Cohen J. D. (2004). The neural bases of cognitive conflict and control in moral judgment. *Neuron* 44 389–400. 10.1016/j.neuron.2004.09.027 15473975

[B22] Greene J. D. Sommerville R. B. Nystrom L. E. Darley J. M. Cohen J. D. (2001). An fMRI investigation of emotional engagement in moral judgment. *Science* 293 2105–2108. 10.1126/science.1062872 11557895

[B23] Greenwald A. G. Banaji M. R. (1995). Implicit social cognition: attitudes, self-esteem, and stereotypes. *Psychol. Rev.* 102 4–27. 10.1037/0033-295x.102.1.4 7878162

[B24] Greenwald A. G. Mcghee D. E. Schwartz J. L. (1998). Measuring individual differences in implicit cognition: the implicit association test. *J. Pers. Soc. Psychol.* 74 1464–1480. 9654756 10.1037//0022-3514.74.6.1464

[B25] Haidt J. (2001). The emotional dog and its rational tail: a social intuitionist approach to moral judgment. *Psychol. Rev.* 108 814–834. 10.1037/0033-295x.108.4.814 11699120

[B26] Hauser M. (2006). *Moral Minds: How Nature Designed Our Universal Sense of Right and Wrong.* New York, NY: Harper Collins.

[B27] Huebner B. Hauser M. D. Pettit P. (2011). How the source, inevitability and Means of Bringing About Harm Interact in Folk-Moral Judgments. *Mind Lang.* 26 210–233. 10.1111/j.1468-0017.2011.01416.x

[B28] Jacobs B. L. Azmitia E. C. (1992). Structure and function of the brain serotonin system. *Physiol. Rev.* 72 165–229. 10.1152/physrev.1992.72.1.165 1731370

[B29] Jonnakuty C. Gragnoli C. (2008). What do we know about serotonin? *J. Cell Physiol.* 217 301–306. 10.1002/jcp.21533 18651566

[B30] Kahneman D. Tversky A. (1984). Choices, values, and frames. *Am. Psychol.* 39 341–350.

[B31] Koenigs M. Young L. Adolphs R. Tranel D. Cushman F. Hauser M. (2007). Damage to the prefrontal cortex increases utilitarian moral judgements. *Nature* 446 908–911. 10.1038/nature05631 17377536 PMC2244801

[B32] Kohlberg L. (1984). *Essays on Moral Development: The Psychology of Moral Development: Moral Stages, Their Nature and Validity*, Vol. 2 New York, NY: Harper & Row.

[B33] Lee J. J. Gino F. (2015). Poker-faced morality: concealing emotions leads to utilitarian decision making. *Organ. Behav. Hum. Decis. Process.* 126 49–64. 10.1016/j.obhdp.2014.10.006

[B34] Lesch K. P. Bengel D. Heils A. Sabol S. Z. Greenberg B. D. Petri S. (1996). Association of anxiety-related traits with a polymorphism in the serotonin transporter gene regulatory region. *Science* 274 1527–1531. 10.1126/science.274.5292.1527 8929413

[B35] Loewenstein G. F. Weber E. U. Hsee C. K. Welch N. (2001). Risk as feelings. *Psychol. Bull.* 127 267–286. 11316014 10.1037/0033-2909.127.2.267

[B36] Lucas B. J. Galinsky A. D. (2015). Is utilitarianism risky? How the same antecedents and mechanism produce both utilitarian and risky choices. *Perspect. Psychol. Sci.* 10 541–548. 10.1177/1745691615583130 26177954

[B37] Luo Q. Nakic M. Wheatley T. Richell R. Martin A. Blair R. J. (2006). The neural basis of implicit moral attitude–an IAT study using event-related fMRI. *Neuroimage* 30 1449–1457. 10.1016/j.neuroimage.2005.11.005 16418007

[B38] Marsh A. A. Crowe S. L. Yu H. H. Gorodetsky E. K. Goldman D. Blair R. J. (2011). Serotonin transporter genotype (5-HTTLPR) predicts utilitarian moral judgments. *PLoS ONE* 6:e25148. 10.1371/journal.pone.0025148 21998637 PMC3187753

[B39] Mazzanti C. M. Lappalainen J. Long J. C. Bengel D. Naukkarinen H. Eggert M. (1998). Role of the serotonin transporter promoter polymorphism in anxiety-related traits. *Arch. Gen. Psychiatry* 55 936–940. 9783565 10.1001/archpsyc.55.10.936

[B40] Mikhail J. (2007). Universal moral grammar: theory, evidence and the future. *Trends Cognit. Sci.* 11 143–152. 10.1016/j.tics.2006.12.007 17329147

[B41] Mrazek A. J. Chiao J. Y. Blizinsky K. D. Lun J. Gelfand M. J. (2013). The role of culture-gene coevolution in morality judgment: examining the interplay between tightness-looseness and allelic variation of the serotonin transporter gene. *Cult. Brain* 1 100–117. 10.1007/s40167-013-0009-x 24404439 PMC3880222

[B42] Munafò M. R. Clark T. Flint J. (2005). Promise and pitfalls in the meta-analysis of genetic association studies: a response to Sen and Schinka. *Mol. Psychiatry* 10 895–897. 10.1038/sj.mp.4001706

[B43] Nosek B. A. Bar-Anan Y. Sriram N. Axt J. Greenwald A. G. (2014). Understanding and using the brief implicit association test: recommended scoring procedures. *PLoS ONE* 9:e110938. 10.1371/journal.pone.0110938 25485938 PMC4259300

[B44] Perkins A. M. Leonard A. M. Weaver K. Dalton J. A. Mehta M. A. Kumari V. (2013). A dose of ruthlessness: interpersonal moral judgment is hardened by the anti-anxiety drug lorazepam. *J. Exp. Psychol. Gen.* 142 612–620. 10.1037/a0030256 23025561

[B45] Phelps E. A. O’connor K. J. Cunningham W. A. Funayama E. S. Gatenby J. C. Gore J. C. (2000). Performance on indirect measures of race evaluation predicts amygdala activation. *J. Cogn. Neurosci.* 12 729–738. 10.1162/089892900562552 11054916

[B46] Piaget J. (1932). *The Moral Judgment of the Child.* Oxford: Harcourt.

[B47] Schinka J. A. Busch R. M. Robichaux-Keene N. (2004). A meta-analysis of the association between the serotonin transporter gene polymorphism (5-HTTLPR) and trait anxiety. *Mol. Psychiatry* 9 197–202. 10.1038/sj.mp.4001405 14966478

[B48] Schmitt M. Baumert A. Gollwitzer M. Maes J. (2010). *The Justice Sensitivity Inventory: Factorial Validity, Location in the Personality Facet Space, Demographic Pattern, and Normative Data.* Berlin: Springer.

[B49] Sen S. Burmeister M. Ghosh D. (2004). Meta-analysis of the association between a serotonin transporter promoter polymorphism (5-HTTLPR) and anxiety-related personality traits. *Am. J. Med. Genet. B Neuropsychiatr. Genet.* 127B 85–89. 10.1002/ajmg.b.20158 15108187

[B50] Spielberger C. D. Gorsuch R. L. Lushene R. E. (1970). *Manual for the State-Trait Anxiety Inventory.* Palp Alto, CA: Consulting Psychologists Press.

[B51] Tomasello M. Vaish A. (2013). Origins of human cooperation and morality. *Annu. Rev. Psychol.* 64 231–255. 10.1146/annurev-psych-113011-143812 22804772

[B52] Walter N. T. Montag C. Markett S. Felten A. Voigt G. Reuter M. (2012). Ignorance is no excuse: moral judgments are influenced by a genetic variation on the oxytocin receptor gene. *Brain Cogn.* 78 268–273. 10.1016/j.bandc.2012.01.003 22296985

[B53] Yang Y. Wang C. Li X. Yu R. Zhang M. Xue M. (2019). The 5-HTTLPR polymorphism impacts moral permissibility of impersonal harmful behaviors. *Soc. Cogn. Affect. Neurosci.* 14 911–918. 10.1093/scan/nsz060 31506681 PMC6847979

[B54] Yoder K. J. Decety J. (2014a). Spatiotemporal neural dynamics of moral judgment: a high-density ERP study. *Neuropsychologia* 60 39–45. 10.1016/j.neuropsychologia.2014.05.022 24905282 PMC4104265

[B55] Yoder K. J. Decety J. (2014b). The Good, the bad, and the just: justice sensitivity predicts neural response during moral evaluation of actions performed by others. *J. Neurosci.* 34 4161–4166. 10.1523/jneurosci.4648-13.2014 24647937 PMC3960462

[B56] Yoder K. J. Decety J. (2018). The neuroscience of morality and social decision-making. *Psychol. Crime Law* 24 279–295. 30766017 10.1080/1068316X.2017.1414817 PMC6372234

[B57] Zhao J. Harris M. Vigo R. (2016). Anxiety and moral judgment: the shared deontological tendency of the behavioral inhibition system and the unique utilitarian tendency of trait anxiety. *Personal. Ind. Diff.* 95 29–33. 10.1016/j.paid.2016.02.024

