# Supplementary material for: The Multifaceted Effects of Serotonin Transporter Polymorphism (5-HTTLPR) on Anxiety, Implicit Moral Attitudes, and Harmful Behaviors
Source: Front Psychol. 2020 Jul 10;11:1521. doi: 10.3389/fpsyg.2020.01521 (PMC7366172; doi:10.3389/fpsyg.2020.01521)
Supplement: Supplementary file 1 [file Table_1.DOCX]

**Supplementary Table 1.** Inter-correlations among dispositional measure of anxiety, justice sensitivity, implicit moral attitudes, and moral permissibility.

| **Correlations** | | | | | | | | | | |
| --- | --- | --- | --- | --- | --- | --- | --- | --- | --- | --- |
|  |  | STAI_S | STAI_T | JS_SelfOriented | JS_OtherOriented | mIAT_DScore | Moral_Personal_Evitable_percent | Moral_Personal_Inevitable_percent | Moral_NonMoral_percent | Moral_Impersonal_percent |
| STAI_S | Pearson Correlation | 1 | .620^**^ | .149 | .013 | .049 | -.096 | .154 | -.191^*^ | -.243^**^ |
|  | Sig. (2-tailed) |  | .000 | .065 | .868 | .563 | .235 | .057 | .017 | .002 |
|  | N | 154 | 154 | 154 | 154 | 141 | 154 | 154 | 154 | 154 |
| STAI_T | Pearson Correlation | .620^**^ | 1 | .387^**^ | .181^*^ | .140 | -.077 | .084 | -.046 | -.060 |
|  | Sig. (2-tailed) | .000 |  | .000 | .025 | .098 | .344 | .299 | .567 | .461 |
|  | N | 154 | 154 | 154 | 154 | 141 | 154 | 154 | 154 | 154 |
| JS_SelfOriented | Pearson Correlation | .149 | .387^**^ | 1 | .383^**^ | .103 | .047 | .100 | -.089 | -.011 |
|  | Sig. (2-tailed) | .065 | .000 |  | .000 | .079 | .492 | .143 | .196 | .876 |
|  | N | 154 | 154 | 305 | 305 | 291 | 214 | 214 | 214 | 214 |
| JS_OtherOriented | Pearson Correlation | .013 | .181^*^ | .383^**^ | 1 | -.007 | -.053 | -.094 | -.063 | -.056 |
|  | Sig. (2-tailed) | .868 | .025 | .000 |  | .909 | .443 | .173 | .361 | .414 |
|  | N | 154 | 154 | 305 | 305 | 291 | 214 | 214 | 214 | 214 |
| mIAT_DScore | Pearson Correlation | .049 | .140 | .103 | -.007 | 1 | .115 | .133 | .033 | -.005 |
|  | Sig. (2-tailed) | .563 | .098 | .079 | .909 |  | .105 | .061 | .638 | .942 |
|  | N | 141 | 141 | 291 | 291 | 291 | 200 | 200 | 200 | 200 |
| Moral_Personal_Evitable_percent | Pearson Correlation | -.096 | -.077 | .047 | -.053 | .115 | 1 | .115 | -.093 | -.063 |
|  | Sig. (2-tailed) | .235 | .344 | .492 | .443 | .105 |  | .092 | .176 | .356 |
|  | N | 154 | 154 | 214 | 214 | 200 | 214 | 214 | 214 | 214 |
| Moral_Personal_Inevitable_percent | Pearson Correlation | .154 | .084 | .100 | -.094 | .133 | .115 | 1 | .102 | .118 |
|  | Sig. (2-tailed) | .057 | .299 | .143 | .173 | .061 | .092 |  | .138 | .084 |
|  | N | 154 | 154 | 214 | 214 | 200 | 214 | 214 | 214 | 214 |
| Moral_NonMoral_percent | Pearson Correlation | -.191^*^ | -.046 | -.089 | -.063 | .033 | -.093 | .102 | 1 | .757^**^ |
|  | Sig. (2-tailed) | .017 | .567 | .196 | .361 | .638 | .176 | .138 |  | .000 |
|  | N | 154 | 154 | 214 | 214 | 200 | 214 | 214 | 214 | 214 |
| Moral_Impersonal_percent | Pearson Correlation | -.243^**^ | -.060 | -.011 | -.056 | -.005 | -.063 | .118 | .757^**^ | 1 |
|  | Sig. (2-tailed) | .002 | .461 | .876 | .414 | .942 | .356 | .084 | .000 |  |
|  | N | 154 | 154 | 214 | 214 | 200 | 214 | 214 | 214 | 214 |
| **. Correlation is significant at the 0.01 level (2-tailed). | | | | | | | | | | |
| *. Correlation is significant at the 0.05 level (2-tailed). | | | | | | | | | | |
